# Third Line Eribulin for Triple-negative Metastatic Breast Ductal Carcinoma Resulting in Extended Progression-free Survival of 57 Months

**DOI:** 10.7759/cureus.6980

**Published:** 2020-02-13

**Authors:** Sukesh Manthri, Purva Sharma, Haider Atheer Mejbel, Sakshi Singal, Devapiran Jaishankar

**Affiliations:** 1 Oncology, East Tennessee State University, Johnson City, USA; 2 Pathology, East Tennessee State University, Johnson City, USA

**Keywords:** eribulin, triple negative breast cancer, metastatic breast cancer

## Abstract

Eribulin is a non-taxane microtubule inhibitor approved for the treatment of metastatic breast carcinoma after two prior chemotherapeutic regimens. We report a patient with extended progression-free survival (PFS) of more than 57 months with metastatic breast carcinoma treated with eribulin in the third-line setting. A 48-year-old lady was diagnosed with stage IIA (pT2N0M0), high grade, triple-negative, invasive ductal carcinoma (IDC) of the left breast on core needle biopsy. She underwent neoadjuvant chemotherapy with adriamycin, and cyclophosphamide followed by a negative sentinel lymph node (SLN) biopsy. Subsequent mastectomy and axillary lymph node dissection revealed a 2.5 cm, high grade, triple-negative IDC with three additional lymph nodes negative for metastatic carcinoma, consistent with the initial diagnosis. Eight months into the surveillance program, the patient developed a 2.8 cm right lower lobe (RLL) lung mass with standard uptake value (SUV) of 27 on positron emission tomography-computed tomography (PET/CT). Core needle biopsy of the lung lesion revealed sheets of poorly differentiated carcinoma, immunophenotypically compatible with the initial diagnosis of breast pathology. She then commenced single-agent paclitaxel in the 1st line metastatic setting with a significant decrease in RLL lung mass to less than 1 cm with an SUV of 1.7 noted. The patient developed progression after seven months and started 2nd line gemcitabine noting initial improvement and subsequent stable disease for a period of 12 months. Eventual progression of RLL lung nodule measuring 2.1 cm with SUV of 10 noted. Initiated 3rd line eribulin with a notable response on imaging studies within three months and with no evidence of disease (NED) on scans over the subsequent 57 months. Eribulin related mild neuropathy superimposed on previous paclitaxel associated grade 2 neuropathy required a 20% eribulin dose reduction. The patient is currently clinically and radiographically stable with plateaued serum tumor markers. Our patient has shown excellent response and tolerance to eribulin with PFS of over 57 months (nineteen times the norm) which is rare.

## Introduction

Triple-negative breast cancer (TNBC) is a term that has been applied to breast cancers that lack expression of the estrogen receptor (ER), progesterone receptor (PR), and human epidermal growth factor receptor 2 (HER2). TNBC tends to behave more aggressively than other types of breast cancer. TNBC accounts for approximately 15% of breast cancers diagnosed worldwide and is more commonly diagnosed in women younger than 40 years. Eribulin is a non-taxane microtubule inhibitor approved for the treatment of metastatic breast cancer after two prior chemotherapeutic regimens. We report a patient with extended progression-free survival (PFS) of more than 57 months with metastatic breast cancer treated with eribulin in the third-line setting [1].

## Case presentation

**Figure 1:**
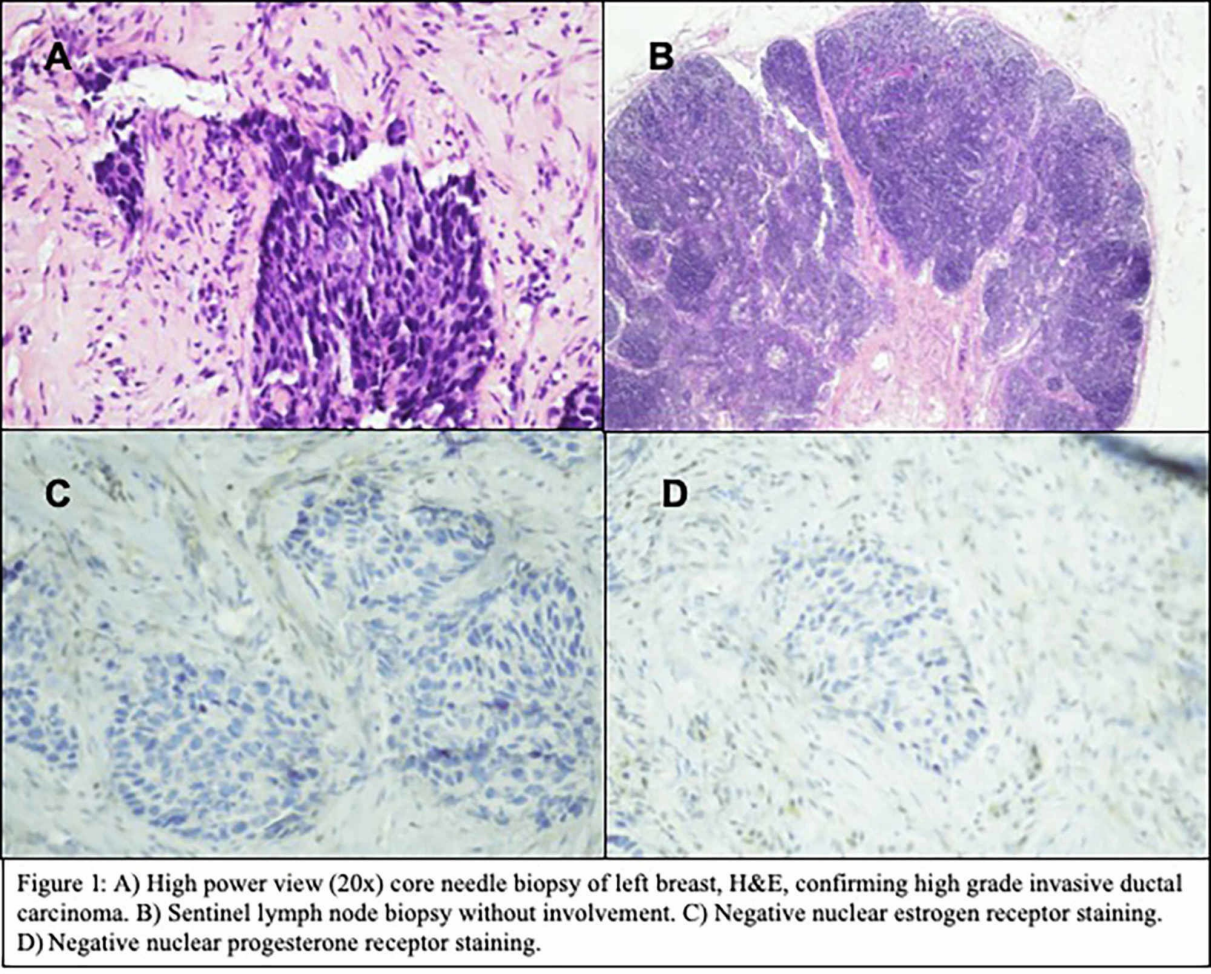
High power view (20x) core needle biopsy of the left breast H&E: hemotoxin and eosin.

A 48-year-old lady was diagnosed with stage IIA (pT2N0M0), high-grade, triple-negative, invasive ductal carcinoma (IDC) of the left breast (Figure 1).

**Figure 2:**
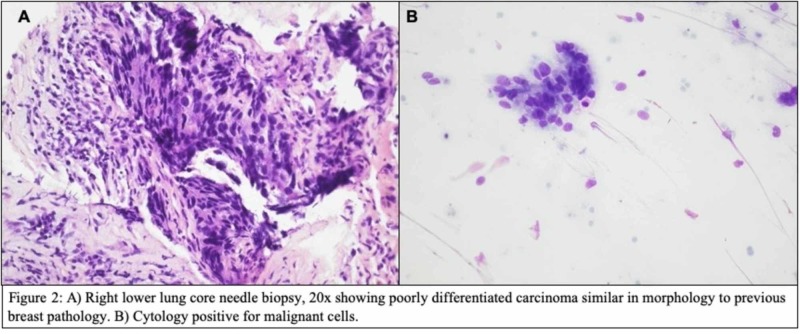
High power view (20x) core needle biopsy of the right lower lobe lung mass

No germline breast cancer type 1 (BRCA1) or type 2 (BRCA2) mutation was detected. She underwent neoadjuvant chemotherapy with adriamycin and cyclophosphamide (AC) followed by a negative sentinel lymph node biopsy. At mastectomy, a 2.5 cm tumor, high grade, triple-negative IDC with three additional lymph nodes negative for metastatic carcinoma was noted. She subsequently pursued further chemotherapy and was treated with two more cycles of AC followed by six cycles of cyclophosphamide, methotrexate, and 5-fluorouracil (CMF) and then transferred care to our cancer center. Eight months into a surveillance program, she developed a 2.8 cm right lower lobe (RLL) lung mass with a standard uptake value (SUV) of 27 along with multiple smaller nodules within the lungs noted on positron emission tomography-computed tomography (PET-CT). A core needle biopsy of the RLL lung mass was consistent with metastatic TNBC with sheets of poorly differentiated carcinoma similar in morphology and immunohistochemical studies to the initial diagnosis of breast pathology (Figure 2).

**Figure 3:**
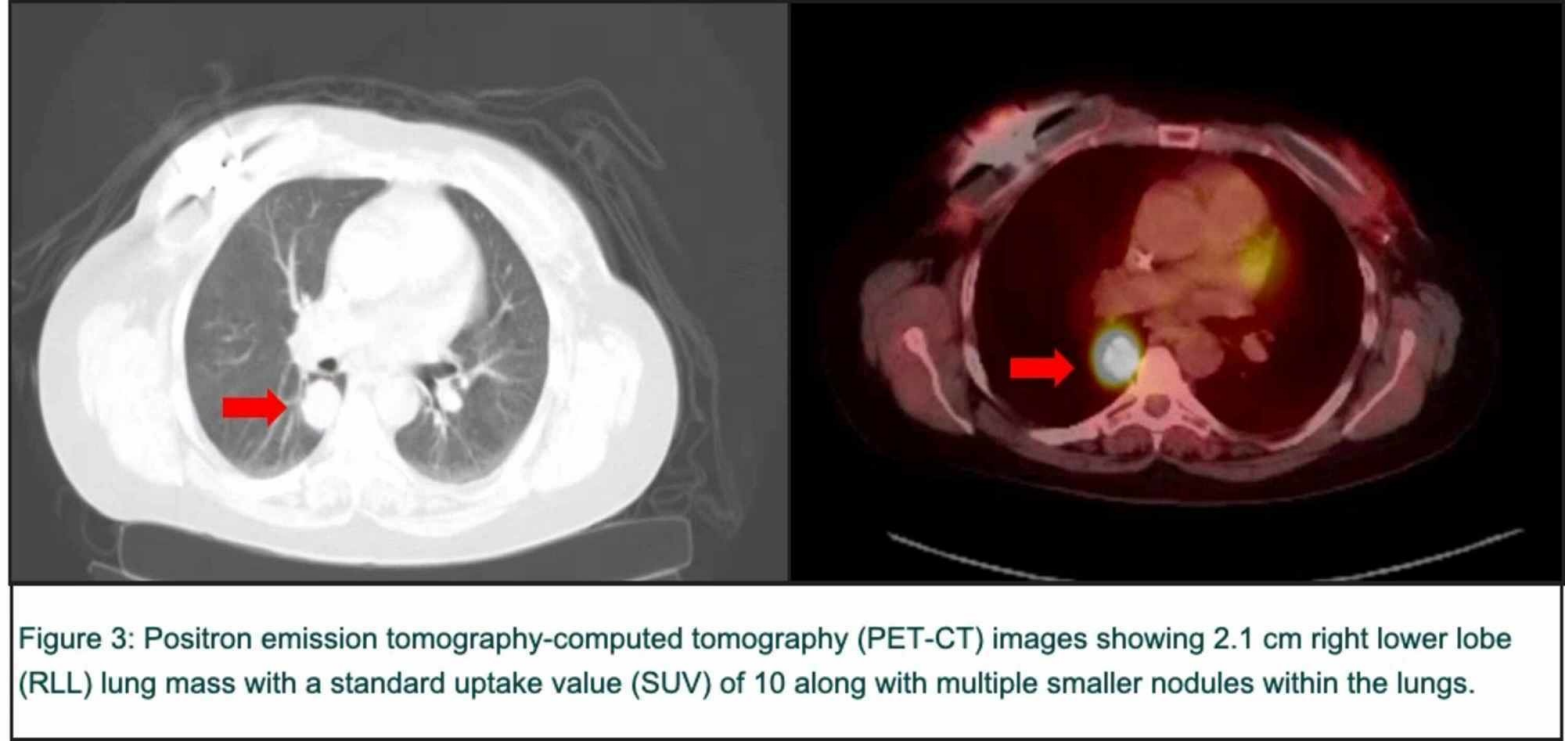
Positron emission tomography-computed tomography (PET-CT) images

Programmed cell death ligand 1 (PD-L1) expressing tumor-infiltrating cells was 0%. She commenced single-agent paclitaxel in the 1st line metastatic setting with a significant decrease in RLL lung mass to less than 1 cm with an SUV of 1.7 and resolution of other sub-centimeter pulmonary nodules. The response lasted seven months. She started 2nd line gemcitabine with initial improvement and subsequent stable disease for a period of 12 months. However, the progression of RLL lung nodule measuring 2.1 cm with SUV of 10 was noted (Figure 3). 

**Figure 4:**
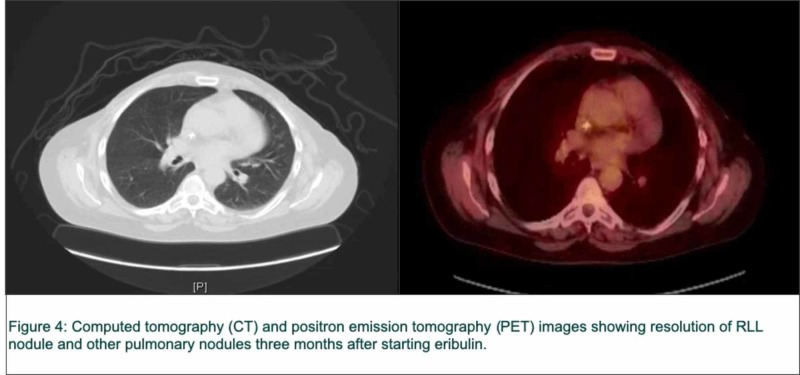
Computed tomography (CT) and positron emission tomography (PET) images RLL: right lower lobe.

3rd line eribulin mesylate 1.4 mg/m2 days 1 and 8 every 21 days was initiated. The latter resulted in a significant response on imaging studies within three months (Figure 4).

**Figure 5:**
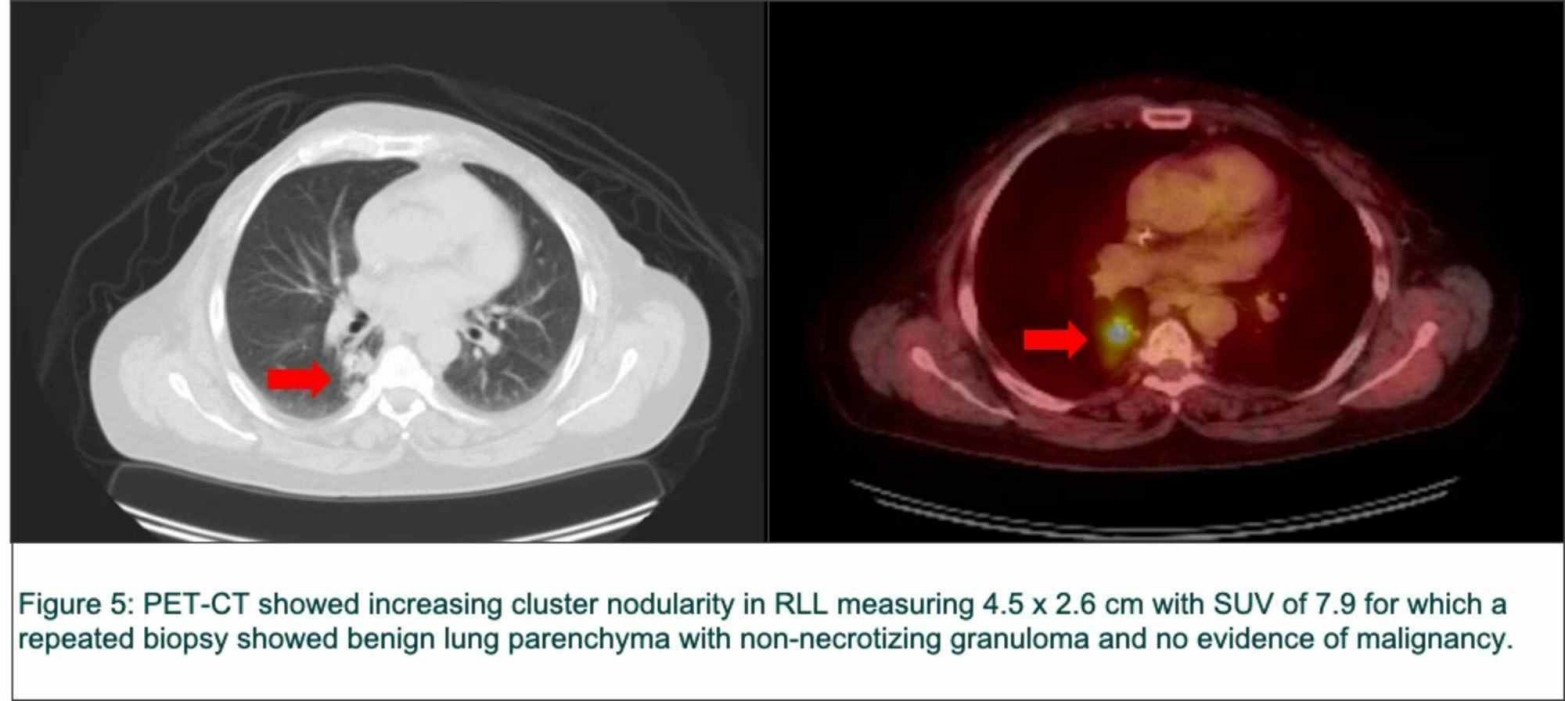
Positron emission tomography-computed tomography (PET-CT) images of the lung RLL: right lower lobe; SUV: standard uptake value.

Approximately 35 months later, a follow-up PET-CT showed increasing cluster nodularity in RLL measuring 4.5 x 2.6 cm with SUV of 7.9 for which a repeated biopsy showed benign lung parenchyma with non-necrotizing granuloma and no evidence of malignancy (Figure 5).

**Figure 6:**
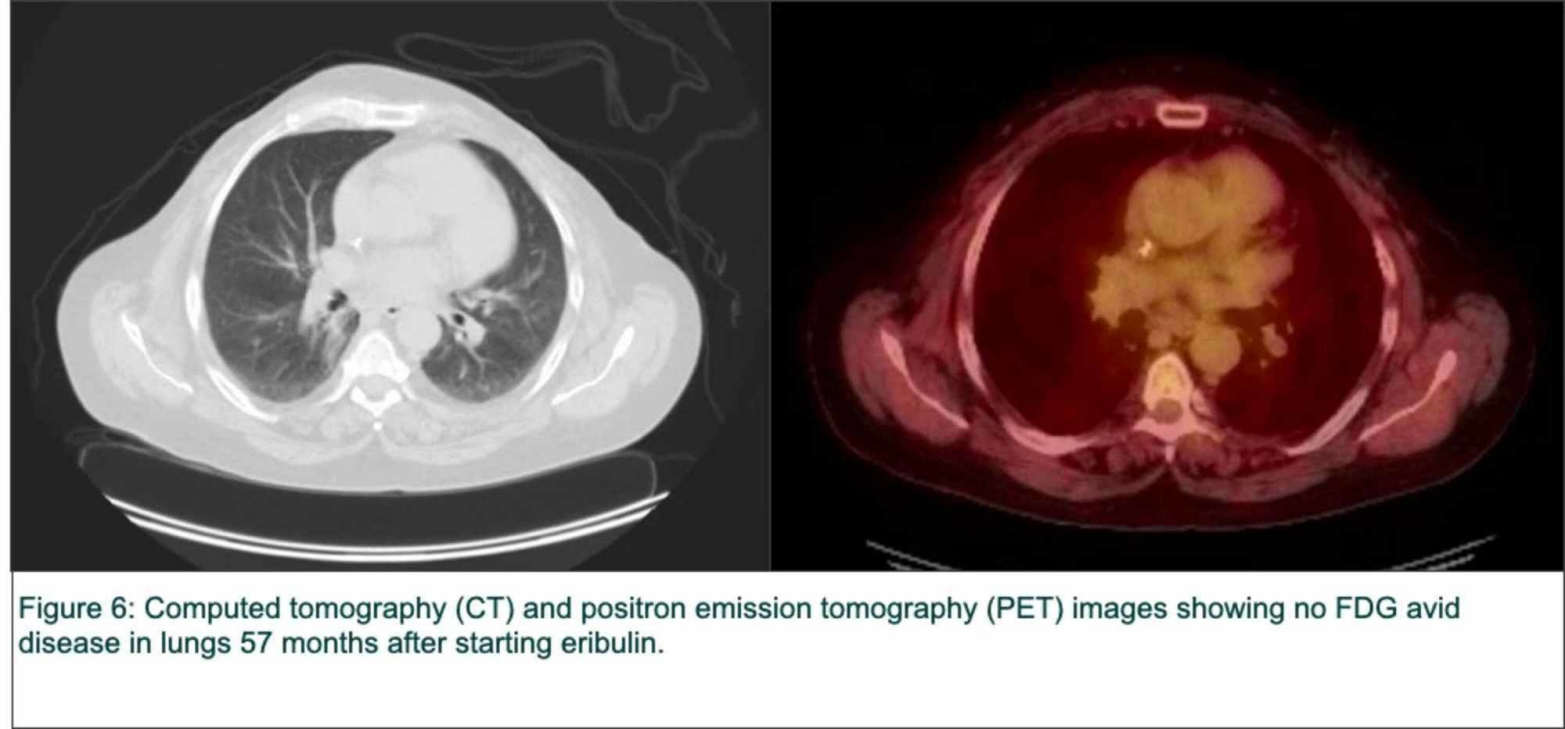
Computed tomography (CT) and positron emission tomography (PET) images of the lungs 57 months after starting eribulin FDG: fluorodeoxyglucose.

She has maintained no evidence of disease (NED) on scans over 57 months (Figure 6).

Currently, she is clinically stable, and her serum tumor markers have plateaued. She has required eribulin 20% dose reduction (1.12 mg/m2 days 1 and 8 every 21 days) on account of neuropathy. 

## Discussion

TNBC is a term referred to as breast cancers that lack the expression of ER, PR, and HER2 on immunohistochemical or molecular studies. TNBC accounts for approximately 15% of all breast cancers diagnosed worldwide that accounts for almost 200,000 cases each year [2]. TNBC is more commonly diagnosed in women younger than 40 years of age and appears to be relatively more common among black women compared with white women [3]. The triple-negative clinical phenotype mostly comprises the basal-like molecular subtype, although there is substantial heterogeneity within TNBCs. In one study of utilizing DNA and RNA profiling of TNBCs, four stable subtypes were identified including luminal androgen receptor, mesenchymal, basal-like immunosuppressed, and basal-like immune-activated [4]. These molecular features may have implications for chemotherapy selection in the future but currently, no such guidelines exist.

TNBC tends to behave more aggressively than other types of breast cancer. The risk of distant recurrence and death peaks approximately three years after diagnosis and declines rapidly thereafter [5]. Chemotherapy has been the mainstay of systemic treatment for TNBC, as endocrine therapy and HER2-directed therapies, are ineffective. There are no prospective data that show combination chemotherapy improves overall survival (OS) compared with single-agent sequential cytotoxic chemotherapy. Combination chemotherapy may be appropriate for those with rapidly progressive visceral disease or crisis (symptomatic lymphangitic lung metastases, bone marrow replacement with associated cytopenias, leptomeningeal carcinomatous, significant liver metastases with associated liver dysfunction), in which the high chance of response is thought to outweigh the risk of toxicity, due to concerns about impending organ dysfunction [6].

Anthracyclines and taxanes are considered the most active chemotherapy agents for metastatic breast cancer, although the increased use of both drugs in adjuvant treatment has prompted the development of other non-cross-reacting agents. Unlike other breast cancer subtypes (i.e., ER-positive, HER2-positive subtypes), there are no approved targeted treatments available for TNBC. Although immunotherapy -in combination with chemotherapy is available for advanced TNBC that expresses PD-L1. For patients without germline BRCA mutation and PD-L1 positive metastatic tumors, the US Food and Drug Administration (FDA) has now approved the use of nab-paclitaxel and atezolizumab. In this randomized trial planned subset analysis of outcomes according to PD-L1-expressing immune effector cells within the tumors, atezolizumab improved both PFS (7.5 versus 5 months), and, importantly, OS (25 versus 15.5 months) [7]. Our patient’s PD-L1 expressing tumor-infiltrating cells were 0% suggesting no benefit of immunotherapy in combination with chemotherapy if she progresses on current third-line chemotherapy.

In patients with no germline mutation and tumors that do not express PD-L1, preferred regimens are anthracyclines (doxorubicin, liposomal doxorubicin), taxanes (paclitaxel), anti-metabolites (capecitabine, gemcitabine), microtubule inhibitors (vinorelbine or eribulin) [8]. Eribulin mesylate (1.4 mg/m2 days 1 and 8 every 21 days) inhibits the polymerization of tubulin and microtubules [9]. In a phase III trial of 762 heavily pretreated patients randomly assigned to treatment with eribulin or other chemotherapy (based on physician's and patient's choice), eribulin improved PFS (3.7 versus 2.2 months) and significantly improved OS (median, 13.1 versus 10.6 months). The objective response rate was 12% with only a 1% complete response (CR) reported in the literature [10]. The second phase III trial was published in 2015 with 1102 patients, who had previously received anthracyclines and taxanes, were randomized in a 1:1 ratio to eribulin or capecitabine. The trial showed eribulin and capecitabine to be equally effective for both PFS and OS. Median PFS was 4.1 months in the eribulin arm in this study [11]. Our patient has shown excellent response and tolerance to eribulin with ongoing PFS of over 57 months (nineteen times the norm) which is rare. 

Case reports with eribulin for heavily pre-treated metastatic breast cancer have been published in the literature. In one series, 15% of patients were TNBC (8/53 patients). In this series median time to progression in TNBC patients was 4.7 months and median OS of 7.43 months [12,13]. To our knowledge, no cases with such lengthy PFS on eribulin chemotherapy, as seen in our patient in a metastatic setting, were reported in the literature. One possible explanation for such a durable response could be low volume disease to start. 

## Conclusions

Historically, eribulin was added to the armamentarium of drugs after being studied in patients who progressed on two or more chemotherapy regimens and PFS was approximately 3.7 to 4.1 months in phase III trials. It is currently unclear if a specific subset of TNBC patients gains maximal benefit. Identification of predictive biomarkers and the concurrent development of diagnostics for these biomarkers are needed. Herein, we present a case of a woman with recurrent triple-negative metastatic breast carcinoma with an extraordinarily lengthy PFS on eribulin chemotherapy.
